# A Photolithographic Approach to Polymeric Microneedles Array Fabrication

**DOI:** 10.3390/ma8125484

**Published:** 2015-12-11

**Authors:** Principia Dardano, Alessandro Caliò, Vincenza Di Palma, Maria Fortuna Bevilacqua, Andrea Di Matteo, Luca De Stefano

**Affiliations:** 1 Institute for Microelectronics and Microsystems, National Council of Research, Via Pietro Castellino 111, Napoli 80131, Italy; alessandro.calio@na.imm.cnr.it (A.C.); luca.destefano@cnr.it (L.D.S.); 2 IMAST Scarl, Piazza Bovio 22, Naples 80133, Italy; vincenza.di-palma@st.com (V.D.P.); maria-fortuna.bevilacqua@st.com (M.F.B.); andrea.di-matteo@st.com (A.D.M.); 3 Department of Physics, University of Napoli “Federico II”, Via Cinthia, Napoli 80100, Italy; 4 STMicroelectronics, via Remo De Feo 1, Arzano, Napoli 80022, Italy

**Keywords:** microneedles, photolithography, polymers

## Abstract

In this work, two procedures for fabrication of polymeric microneedles based on direct photolithography, without any etching or molding process, are reported. Polyethylene glycol (average molecular weight 250 Da), casted into a silicone vessel and exposed to ultraviolet light (365 nm) through a mask, cross-links when added by a commercial photocatalyzer. By changing the position of the microneedles support with respect to the vessel, different shapes and lengths can be achieved. Microneedles from a hundred microns up to two millimeters have been obtained just tuning the radiation dose, by changing the exposure time (5–15 s) and/or the power density (9–18 mW/cm^2^) during photolithography. Different microneedle shapes, such as cylindrical, conic or lancet-like, for specific applications such as micro-indentation or drug delivery, are demonstrated.

## 1. Introduction

By exploiting standard technologies and facilities in the fabrication of micro and nano electro mechanical systems, a new generation of biomedical devices is currently realized and continuously developed. Physical and chemical characteristics of these devices can be finely tuned on a very small scale, down to few nanometers, gaining popularity in biomedicine field over the last decade [1,2]. Microneedles (MNs) are key components of some biomedical systems, often acting as interface between the device and the body of the patient. MNs shape, length, density, tip model, as well as the material of which they are made of, can be very different, depending on the specific application considered [1,2,3,4,5]. MNs based devices can be exploited in drugs and genes delivery [3,4], in physiologic fluids extraction [5], in cell therapy [6] and also in diagnostics [7]. Due to well-established microelectronic fabrication technologies, silicon, and silicon related materials, such as porous silicon, silicon nitrides and silicon oxide, is one of the most used and lot of papers and patents can be found in literature [8]. On the other hand, silicon is fragile and definitely not a biocompatible material, since it can cause local inflammations or silicosis, so that even in low invasive devices it could result unhealthy. As alternative materials, polymers have been extensively used in many published works to overcome intrinsic limitations of silicon: one of the most used is Poly Dimethyl Siloxane (PDMS), which is the main component in microfluidic circuits and also due to its biological compatibility [9]. Moreover, it has a long history as non-toxic and non-immunogenic polymer for several drug delivery applications [2]. Many other biodegradable polymers have been extensively employed in MNs fabrication for drugs delivery application [10,11,12,13,14].

Hydrogels are attractive materials in fabrication of biodevices, since a hydrated gel provides an excellent matrix for encapsulation of functional biological molecules, such as enzymes and peptides [9,10,15,16]. Main advantages are briefly summarized in the following: (a) device miniaturization by photolithographic patterning; (b) development of biocompatible tissue/sensor interface; (c) near-physiological conditions that minimize protein denaturation helping them to carry out their full biological functions; and (d) three-dimensional geometry of hydrogels enables them to contain large quantity of sensing reagent, thereby increasing their signal-to-noise ratio and sensitivity [16,17].

Poly(ethylene glycol) diacrylate (PEGDA) is a biologically inert polymer that rapidly hardens at room temperature in presence of a photo-initiator on exposure to ultraviolet (UV) light, so that it is possible to fabricate PEGDA MNs by standard photolithographic process. PEGDA is hydrophilic, elastic and can be customized to include a variety of biological molecules or inorganic nanoparticles [15,16,17,18,19]. However, the type of target materials and size are critical factors in determination of microneedles applicability. Tailoring of size, shape and tip of MN is highly desirable.

Generic photolithographic processes in microelectronic technology require that a substrate is covered by spin-coating a liquid photosensitive polymer, called photoresist, which is later hardened by a thermal process. Then, the photoresist is exposed to ultraviolet radiation through a mask, which reproduces the design of the structure to be realized. In our case, the sketch on the mask is the array of circles that constitute the base of needles. UV light creates or destructs “cross-link” bonds of the polymer, in case the resist is “negative” or “positive”, respectively. In this way, the solubility of the exposed resist is drastically different from that in the shade of mask. By dipping the sample in the developing solution, the portion of the photoresist exposed (or not exposed, depending on whether the photoresist is negative or positive, respectively) is removed. As the last step, the so-called etching step, the drawing made of photoresist is then reproduced on the substrate with wet or dry chemical attack [20].

Kochhar *et al.* [21], proposed a method for MNs array fabrication based on a single step process, realizing MNs with truncated cone shape and variable length upper to 1 mm.

In this work, we proposed two procedures (Method 1 and Method 2 in the following) of direct photolithography, where the mixture of PEGDA and DAROCUR^©^ (BASF, Ludwigshafen am Rhein, Germany) has been used just like an ordinary photoresist and the etching step was not longer required to transfer the design on the substrate. The present photolithographic approach to fabricate PEGDA MNs, using microelectronic fabrication facility and skills, allows the realization of MNs with different shape, length and tip by changing the exposure parameters and the position of the support. The processes provide to a wide range of MN types, useful either for epidermis or dermis targeting. In fact, the needle morphological and dimensional features can be decided by adjusting the exposure parameters, depending on the specific application.

## 2. Experimental Section

### 2.1. Materials

All chemicals are commercially available and used as received. We use a solution of PEGDA (number average molecular weight, *M*n = 250, Sigma Aldrich, St. Louis, MO, USA) mixed with 2-Hydroxy-2-methyl-1-phenyl-propan-1-one (DAROCUR^©^ 1173, BASF) at 2% (*v*/*v*). The DAROCUR^©^ is a liquid photo-initiator which is used to initiate the photo-polymerization of chemically unsaturated pre-polymer solution [15,16].

Polyethylene naphtalate (PEN) Q83 (Teonex Q83, DuPont Teijin Films, Wilton, UK) is a hard sheet of plastic-like acetate in form of a transparent sheet with thickness of 125 μm.

### 2.2. Direct Photolithographic Methods

**Figure 1:**
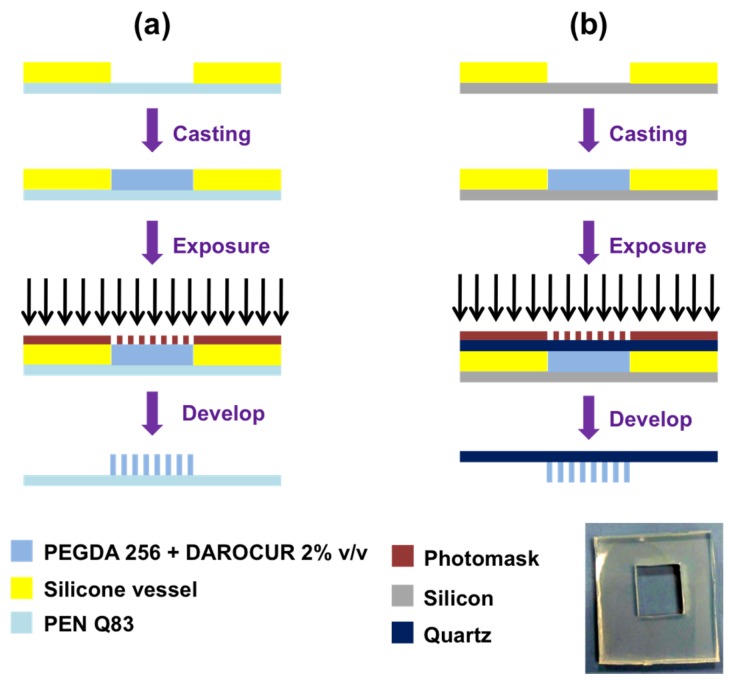
Sketches (not to scale) of the photolithographic process for the microneedles (MNs) fabrication by Method 1 (**a**) and Method 2 (**b**). Inset: the silicone vessel has a close cavity form.

In this work, we proposed two procedures (Method 1 and Method 2) of direct photolithography, where the mixture of PEGDA and DAROCUR^©^ has been used as photoresist and the etching step was not longer required to transfer the design on the substrate (see the flow charts reported in Figure 1). The PEGDA, initially in liquid state, was directly hardened by UV exposure, thus it was used as “negative” photoresist. The exposure step has been performed with MA6/BA6 mask aligner (by SÜSS MicroTec AG, Garching, Germany) at 365 nm. The photolithographic mask was made of two arrays of transparent circles: the diameters of circles were 400 and 250 μm in size for arrays “A” and “B”, respectively. In both methods, the liquid mixture was casted into a silicone vessel, whose volume is about 0.4 × 1.0 × 1.5 cm^3^ (inset picture in Figure 1). The vessel has been fabricated by shaping with a cutter a medical grade silicone sheet (71-MED-40D-187 by CS Hyde Company, Lake Villa, IL, USA) in close cavity form.

#### 2.2.1. Method 1

In this configuration, sketched in Figure 1a, a layer of PEN Q83 was used as substrate during the process in order to obtain a flexible support for the hardened needles (Figure 1a). PEN was placed at the bottom of the vessel as a solid base. Since PEN has good adhesion to the silicone walls of the vessel, it was possible to cast the liquid without losses. The silicone vessel has a dual function, *i.e.*, it is both a container for the liquid resist and a spacer able to define the height of the needles. The exposure to UV light was made by a mask-aligner with soft contact or in close proximity to prevent contamination of the mask. Finally, samples were developed in deionized water and dried with nitrogen.

Different deposition methods of the liquid PEGDA in several processes have been exploited in order to improve the length and the shape of MNs.

#### 2.2.2. Method 2

In this case, a quartz layer was on top of the vessel and was used as substrate for the hardened needles, once reversed (Figure 1b). The back-side (not-polished) of a silicon wafer was used as base of the vessel during the process in order to minimize back reflection effects at the bottom of the vessel (Figure 1b). To improve the adhesion to the silicone walls of the vessel, the silicone bottom surface was wetted by isopropyl alcohol, whose high vapor tension prevented lateral losses for short exposure times. The vessel was filled with PEGDA solution, in order to have direct contact between the quartz layer and the PEGDA solution. In addition, in this case, the silicone had the dual function of container and spacer to define the maximum height of the needles. The exposure is made by a mask-aligner in contact to the quartz layer. Finally, the samples were developed in deionized water and were dried by nitrogen.

The dependence of MNs height and shape on the dose of radiation, *i.e.*, the energy density, has been studied by changing the power density of the UV source (18, 15, 12 and 9 mW/cm^2^) and the exposure time (5, 7.5, 10, 12.5, 15 s). Moreover, both the arrays “A” and “B” have been investigated.

### 2.3. Characterization

#### 2.3.1. Image Analysis

For MNs higher than 300 μm, the length was measured by image analysis. The average height of the MNs arrays was evaluated by images captured by the camera of a water contact angle analyzer (WCA, 1000 C Class, First Ten Angstroms, Portsmouth, VA, USA).

Some MN has been removed by the quartz substrate and measured aside. Then, the image has been performed and analyzed by Leica DM6000 M microscope (Leica, Wetzlar, Germany) equipped by Leica DFC 280 digital camera system (Leica).

#### 2.3.2. Scanning Electron Microscopy

Micro and nano features of MNs, such as surface roughness and curvature radius of the tip, have been evaluated from images by a scanning electron microscopy (SEM) and by the optical microscope.

SEM images were performed at 5 kV accelerating voltage and 30 μm wide aperture by a field emission scanning electron microscope (Carl Zeiss NTS GmbH 1500 Raith FESEM, Carl Zeiss, Oberkochen, Germany). Secondary emission detector has been used. The polymeric MNs were been gold coated to improve the conductivity and, thus, the definition of the SEM images. In order to evaluate the porous structure, the gold has been partially removed by a single MN.

#### 2.3.3. Mechanical Tests

Mechanical properties of PEGDA based MNs have been characterized by measuring the indentation hardness of the MN tip by means of force/distance (F/D) spectroscopy tool of the atomic force microscope (AFM, 70× model, Park Systems, Santa Clara, CA, USA). The F/D spectroscopy tool supports the acquisition of force *vs.* distance curve, which is a plot of the force between the AFM tip and the sample as function of their relative distance (the off set position is determined by the set point value). In particular, the indentation hardness of the MN tip has been compared with the indentation hardness of a Parafilm^®^ (Bemis, Oshkosh, WI, USA) layer , used as artificial skin as reported in [22], where mechanical properties of Parafilm^®^ have been proved to be very close to that of pigskin. Moreover, penetration test have been performed by using eight Parafilm^®^ layers folded on their self; images of the first layer have been captured both at optical microscope and by the camera of the WAC.

The water content of MNs has been estimated by thermogravimetric analysis (TGA) in order to evaluate the polymer decomposition, as also reported in ref. [23]. The thermogram was carried out by PerkinElmer Pyris 1 (PerkinElmer, Waltham, MA, USA) and was recorded from 30 to 500 °C under nitrogen flow at a rate of heating 10 °C/min.

The samples have been immersed in Phosphate Buffer PBS (10 mM, pH 7.4) at 37 °C. At fixed times (24, 48, 72 and 144 h), samples have been recovered and analyzed after removing of water excess.

The average water loss was calculated as the loss in weight at 100 °C on three samples at each fixed time.

## 3. Results and Discussion

PEGDA is well known in literature to be a polymer in nature, characterized by hydrophilic behavior even after polymerization, and capability to mimic the role of extracellular matrix of living tissues [24,25]. We choose the UV polymerization of PEGDA (Molecular weight, MW 250) promoted by a commercial photoinitiator, to obtain MNs with a proper Young’s Modulus for indentation. Infrared spectroscopy investigation (data not reported here), before and after UV exposure, demonstrated that more than 95% *v*/*v* of PEGDA crosslinked, and the excess of unreacted product was removed by water rinsing. The MNs, fabricated by both of the two methods described here, are also hydrophilic in physiological medium (PBS at 37 °C).

### 3.1. Method 1

Preliminary investigations were focused on testing two different depositions of PEGDA on the substrate, namely by casting it in a silicone vessel or directly spinning it, once fixed the power density of the lamp (18 mW/cm^2^) and the exposure time (1000 s) in the mask aligner instrument. Results are summarized in Table 1.

**Table 1:** Fabrication parameters and results for Method 1.

Sample Name	Dose (J/cm^2^)	Deposition Method	Array Type	Height (μm)	Shape
1B1	18	Casting in vessel (2 mm)	B	150	cylinder
2B1	18	Spinning (400 rpm)	B	70	cylinder
3B1	18	Spinning (800 rpm)	B	20	cylinder

The MNs length depended on the deposition methods: results in case of spinning deposition suggested that it was impossible to obtain MNs high enough to overcome the stratum corneum, *i.e.*, longer than 150–200 μm. The best result, in this view, was achieved by casting the PEGDA solution in the silicone vessel. Nevertheless, the shape was not the one desired and exposure was time-consuming, and, as a consequence, expensive in cost. In all experiments, truncated cones, very similar to cylinders without sharp tips were obtained: this shape could fit some microfluidic applications but is not like real needles.

### 3.2. Method 2

In Method 2, different vessel geometry has been experimented for both “A” and “B” array: in this way, MNs were produced faster with respect to Method 1. Moreover, changing the dose of exposure radiation enabled different lengths and shapes.

The length of MNs and shape type are reported in Table 2, where the power density of the UV source is fixed to his maximum, *i.e*., 18 mW/cm^2^, and the exposure time are 5, 7.5, 10, 12.5, 15 s. Moreover, results using both arrays “A” and “B” are reported (Table 2).

**Table 2:** Fabrication parameters and results for Method 2 with power density 18 mW/cm^2^.

Sample Name	Exposure Time (s)	Array Type	Height (μm)	Shape
1A2	5	A	1340	cone
2A2	7.5	A	1430	lance
3A2	10	A	1450	lance
4A2	12.5	A	1700	lance
5A2	15	A	2240	lance
1B2	5	B	1230	cone
2B2	7.5	B	1430	cone
3B2	10	B	1690	lance
4B2	12.5	B	1700	lance
5B2	15	B	2000	lance

**Figure 2:**
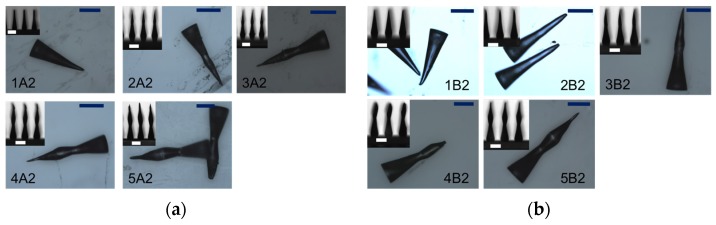
(Scale bar is 500 μm) MNs have been removed by the quartz substrate and images have been captured by a standard optical microscope. The labeling follows the sample names indicated in Table 2. In the insets, images of MNs arrays on the quartz substrate have been captured by the water contact angle analyzer (WCA) camera. (**a**) MNs array obtained by mask array “A”; (**b**) MNs array obtained by mask array “B”.

In Figure 2, the collected images of MNs (for samples 1A2, 2A2, 3A2, 4A2, 5A2 and 1B2, 2B2, 3B2, 4B2, 5B2) arrays, and also for single elements, are shown.

In this geometry, exposure times were considerably lower than the ones used in Method 1 (fixed at 1000 s). The height of MNs ranged between 1200 and 2200 μm and increases as the exposure time increases, and then the dose increases. MNs fabricated with array A (400 μm diameter) were higher than MNs by array B (250 μm) for equal exposure time. However, the “lancet” shape was demonstrated for both arrays, even with a slight increasing of the exposure time. The effect can be explained by considering that the cross-linked PEGDA is denser than the unexposed one, so that also its refractive index is higher. The ultraviolet light, coming from the mask aligner lamp, is thus confined inside the cross-linked PEGDA (as it happens to light confined in an optical fiber core), so that the light intensity is higher than elsewhere inside the forming MNs. In this way, a variety of MNs shapes and lengths can be finely tuned by changing the process parameters, *i.e.*, the power radiation of the lamp and the exposure time. Moreover, the polymerization process induces a typical nanoporosity of the PEGDA matrix, as well as also in other polymers, that is a very important feature for biomedical applications: MNs could be used as a probe to sense human fluids or deliver biochemical substances, previously absorbed inside the tip, after skin or tissue indentation.

**Figure 3:**
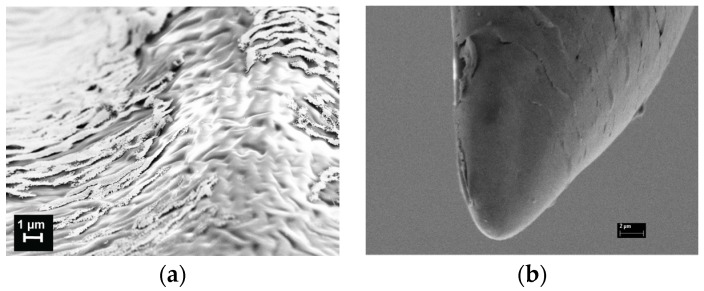
Scanning electron microscopy (SEM) images of a single MN gold coated: (**a**) the gold was partially removed in order to show the roughness on surface; and (**b**) the tip of the MN has a curvature radius of about 2 μm.

In Figure 3, we report SEM images of sample 1B2, as an example, where the MN surface roughness was estimated of about 1 μm in value (Figure 3a) and the curvature radius of about 3 μm was measured (Figure 3b).

In order to prove the fabrication of smaller MNs, power radiation of the mask aligner UV lamp, at a fixed exposure time (7.5 s), has been decreased. Results are reported in Table 3 for the MNs family A2 (array A, Method 2) and B2 (array B, Method 2), as examples.

**Figure 4:**
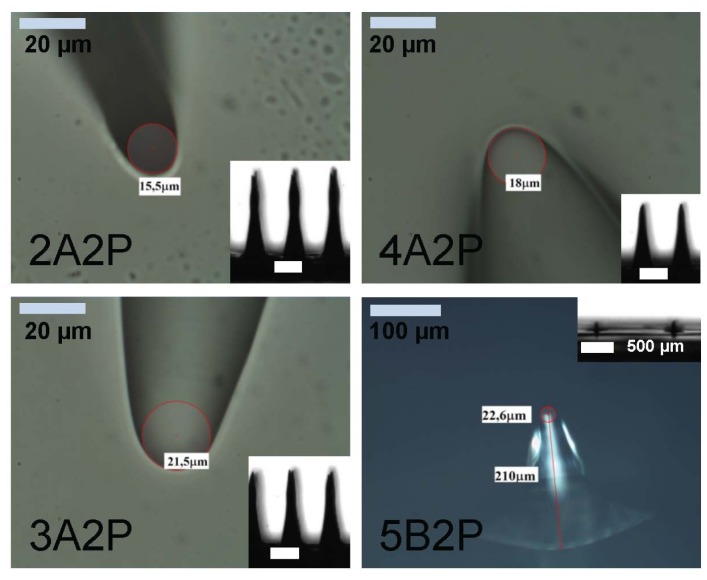
Images of the MNs tips: red circles underline the tip curvatures; diameter of curvature is indicated. The labeling follows the sample names in Table 3. 2A2P, 3A2P and 4A2P samples have been exposed through mask “A” at 15, 12 and 9 mW/cm^2^ power radiation, respectively (scale bar is 20 μm). 5B2P has been exposed through mask “B” at 9 mW/cm^2^ (scale bar is 100 μm). In the insets, images of MNs arrays on the quartz substrate captured by the WCA camera are shown (scale bar is 500 μm).

In Figure 4, the collected images of MNs realized with different power radiation are shown. It is worth noting that optical powers of 15 and 18 mW/cm^2^ gave the same result in term of MN length (about 1450 μm, 7.5 s of time exposure) that implies a sort of saturation effect, which limits this kind of fabrication process.

**Table 3:** Fabrication Parameters and results for Method 2 by changing the exposure optical power.

Sample Name	Power Radiation (mW/cm^2^)	Array Type	Exposure Time (s)	Height (μm)	Diameter (μm)
1A2P	18	A	7.5	1450	4
2A2P	15	A	7.5	1450	15.5
3A2P	12	A	7.5	1180	21.5
4A2P	9	A	7.5	940	18.0
5B2P	9	B	7.5	210	22.6

The fabrication procedure optimized in Method 2 allows the production of sharp MNs, which can be useful for skin penetration or indentation in sensing applications [26,27], and “lancet” shaped MNs, potentially useful in drug delivery and mechanical interlocking of tissues [28]. Moreover, considering that during the crosslinking process drugs can be expelled from a gel matrix, encapsulation efficiency decreases by using longer UV exposure. Then, a fast process is desirable.

**Figure 5:**
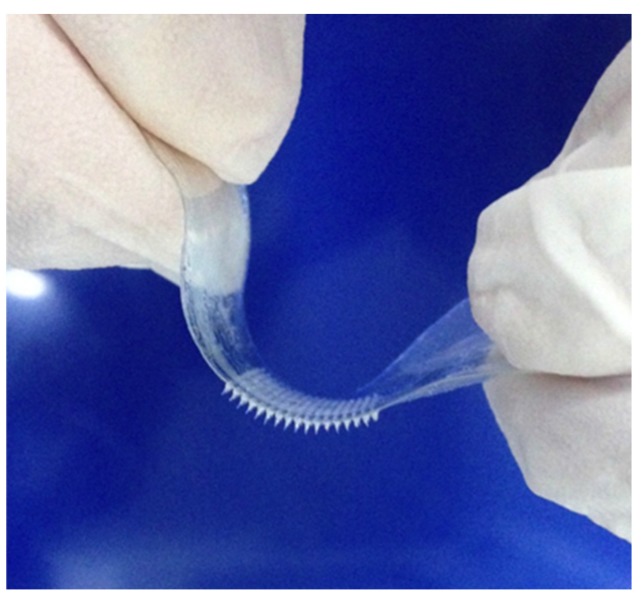
Photo of MNs array on poly(ethylene glycol) diacrylate (PEGDA). The structures are bended by fingers without breaking or damaging the MNs.

Fabrication processes were firstly developed on quartz support, but same procedures were repeated using a layer of PEGDA covering the quartz surface, in order to fabricate the MNs array on a flexible support. In the photograph reported in Figure 5, a MNs array on PEGDA support, easily removed from the quartz by tweezers, is shown. The adhesion of the array on the plastic support is perfect and the structure can be stretched and squeezed by fingers without breaking or damaging the MNs.

**Figure 6:**
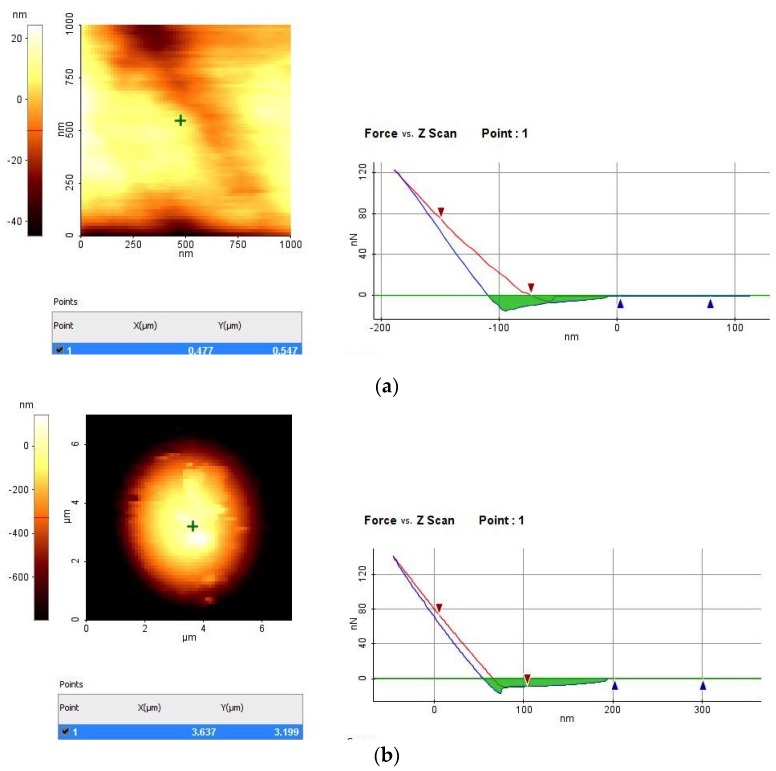
Atomic force microscope (AFM) relative indentation hardness measurements of a Parafilm^®^ layer (**a**) and a MN tip (**b**).

Since MNs penetration into the skin is a major issue, mechanical properties of MNs have been tested with Parafilm^®^ layers, used as artificial skin very similar to pig derma, as reported in ref. [22]. Figure 6 shows the relative indentation hardness of a Parafilm^®^ layer (a) compared with the indentation hardness of the central point of a single tip (b). The force spectroscopy plot shows a greater slope, and thus a greater hardness of the MN tip with respect to the artificial skin. This result is essential in quantifying the possibility of a MN in tissue penetration [22].

**Figure 7:**
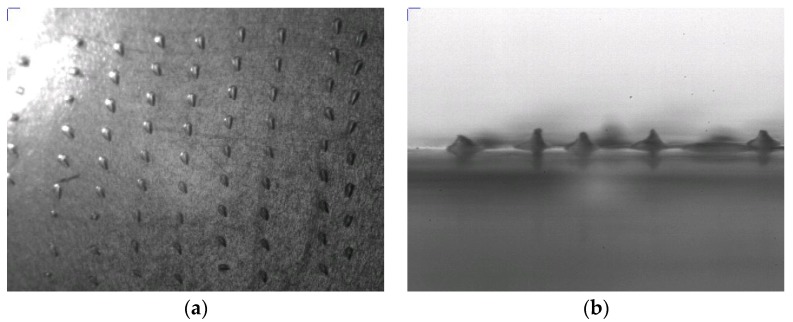
Water contact angle analyzer (WCA) images of the first Parafilm^®^ layer after reversing: (**a**) tilted image and (**b**) lateral view.

**Figure 8:**
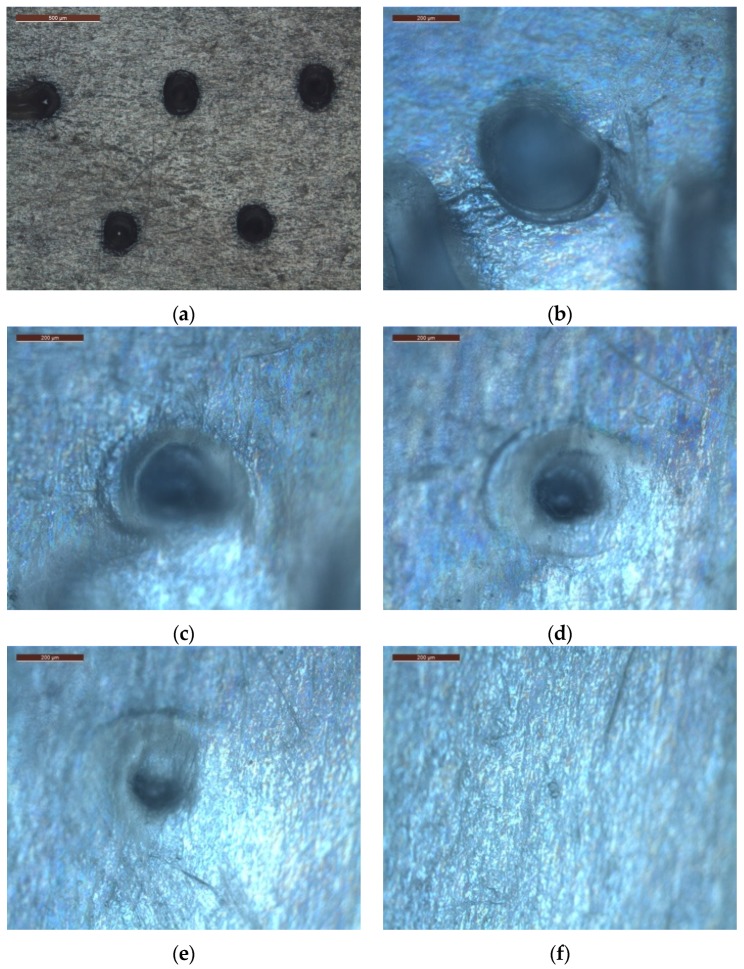
Optical microscope images: (**a**) (the bar is 500 μm) top view in brightfield of the first Parafilm^®^ layer; and (**b**–**f**) (the bar is 200 μm) top view in differential interference contrast (DIC) mode of Parafilm^®^ layers 1–5, respectively.

The penetration tests, as described in ref. [22], have been performed by using eight-folded Parafilm^®^ layer. Figure 7 and Figure 8 show the result of penetration tests. In particular, photographic images of the first layer, once separated from the others, reversed and photographed in tilted (45°) view (a) and in lateral (90°) view (b) are reported: it is clear that the MNs penetrated the first layer and impressed their shape by molding the surface.

In Figure 8, top views of the first five layers of the artificial skin are shown. In particular, in Figure 8b–f, the holes due to the same MN have been imaged from the first to the fifth layer. Since each Parafilm^®^ layer is 160 μm thick, the MN 1340 μm long penetrates at least 640 μm, proving a good penetration strength. Shorter MNs gave better results in Parafilm^®^ penetration, whereas higher MNs tend to bend on themselves without breaking (data not shown here).

We also investigated the capability of the PEGDA-MNs to properly swell on exposure to water solution. The water content in MNs was estimated by thermogravimetric analysis (TGA).

Samples were immersed in PBS solution 10 mM, pH 7.4, at 37 °C. At fixed times (24, 48, 72 and 144 h), samples were dried and analyzed.

**Figure 9:**
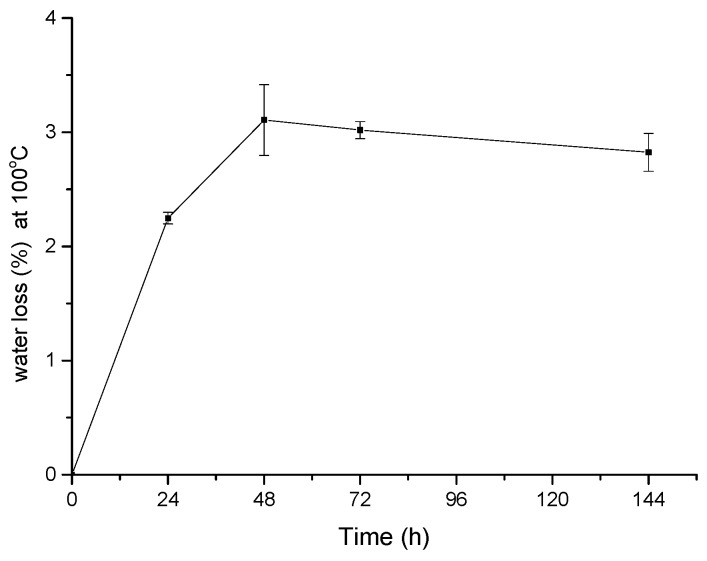
Water loss at 100 °C of microneedles after immersion in buffer solution at fixed time. Experimental points are reported together with their standard deviation.

As shown in Figure 9, the water content absorbed by MNs array increased up to 3% in weight during the first 48 h, then the effect saturated.

**Figure 10:**
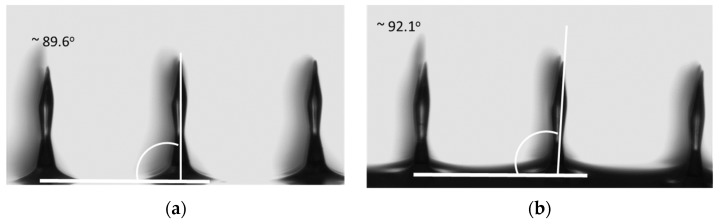
Picture of microneedles before (**a**) and after 48 h (**b**) immersion in buffer solution. We show the MNs normal axis to the base line and tilt angle. The picture and the tilt angle are obtained using DataPhysics Instruments OCA20 (DataPhysics corporation, Filderstadt, Germany).

The water absorption caused a weak deformation of MNs shape, as it can be seen in Figure 10, where a small inclination of MNs can be observed: 89.6° *vs.* 92.1° tilt angles before and after immersion, respectively. This result confirmed that the PEGDA based MNs demonstrated a great stability in time as well as in physiologic environment, and could be exploited in continuous monitoring application.

Finally, any appreciable degradation of MNs array in terms of mechanical properties after a period of 3–5 days could not be found. In agreement with our results, Browning *et al.* [29] referred that a 10 wt % solution of PEGDA in sterile water exposed at UV light for 6 min had very low degradation after four weeks in terms of swelling ratio and modulus, and higher degradation occurred after 12 weeks during “*in vivo*” experiments. However, the proposed MNs are based on strongly cross-linked PEGDA (which means very low water content), as proved by the small water content after 144 h in buffer PBS at 37 °C. Than, the reactivity of PEGDA in proposed MN is lower than in water solution.

## 4. Conclusions

In this work, we exploited two similar fabrication procedures (Method 1 and Method 2) for producing polymeric MNs arrays on both hard and flexible supports. MNs were made of PEGDA, that allows casting and direct polymerization, thus eliminating etching step in the fabrication process and drastically decreasing production cost.

Method 1 (support at bottom) enables the fabrication of MNs with cylindrical shape and heights up to 150 μm. Method 2 (support at the top) enables conical and lancet shapes with tunable highness in the range from 150 to 2240 μm, by simply changing the dose of exposure radiation in terms of time and power. Method 2 resulted faster than Method 1, since exposure time was less by two orders of magnitude. Finally, the presented methods can be easily integrated in a microelectronic fabrication process, and due to their properties this kind of MNs can be the perfect interface between the patient body and a microelectronic device for health monitoring.

## References

[1] Ashraf M.W., Tayyaba S., Afzulpurkar N. (2011). Micro electromechanical systems (MEMS) based microfluidic devices for biomedical applications. Int. J. Mol. Sci..

[2] Pasut G., Veronese F.M. (2007). Polymer-drug conjugation, recent achievements and general strategies. Progress Polym. Sci..

[3] Henry S., Mcallister D.V., Allen M.G., Prausnitz M.R. (1998). Microfabricated microneedles: A novel approach to transdermal drug delivery. J. Pharm. Sci..

[4] Valdés-Ramíreza G., Windmiller J.R., Claussen J.C., Martinez A.G., Kuralay F., Zhou M., Zhou N., Polsky R., Miller P.R., Naravan R. (2012). Multiplexed and switchable release of distinct fluids from microneedle platforms via conducting polymer nanoactuators for potential drug delivery. Sens. Actuators B.

[5] Mukerjee E.V., Collins S.D., Isseroff R.R., Smith R.L. (2004). Microneedle array for transdermal biological fluid extraction and *in situ* analysis. Sens. Actuators A Phys..

[6] Donnelly R.F., Morrow D.I. J., McCarron P.A., Woolfson A.D., Morrissey A., Juzenas P., Juzeniene A., Iani A., McCarthy H.O., Moan J. (2008). Microneedle-mediated intradermal delivery of 5-aminolevulinic acid: Potential for enhanced topical photodynamic therapy. J. Controll. Release.

[7] Miller P.R., Skooga S.A., Edwards T.L., Lopez D.M., Wheeler D.R., Arango D.C., Xiao X., Brozik S.M., Wangc J., Polsky R. (2012). Multiplexed microneedle-based biosensor array for characterization of metabolic acidosis. Talanta.

[8] Wilke N., Mulcahy A., Ye S.R., Morrissey A. (2005). Process optimization and characterization of silicon microneedles fabricated by wet etch technology. Microelectron. J..

[9] Tan J.L., Tien J., Pirone D.M., Gray D.S., Bhadriraju K., Chen C.S. (2003). Cells lying on a bed of microneedles: An approachto isolate mechanical force. Proc. Natl. Acad. Sci..

[10] Vecchione R., Coppola S., Esposito E., Casale C., Vespini V., Grilli S., Ferraro P., Netti P.A. (2014). Electro-drawn drug-loaded biodegradable polymer microneedles as a viable route to hypodermic injection. Adv. Funct. Mater..

[11] Sun W., Araci Z., Inayathullah M., Manickam S., Zhang X., Bruce M.A., Marinkovich P.M., Lane A.T., Milla C., Rajadas J. (2013). Polyvinylpyrrolidone microneedles enable delivery of intact proteins for diagnostic and therapeutic applications. Acta Biomater..

[12] Kim M.Y., Jung B., Park J.H. (2012). Hydrogel swelling as a trigger to release biodegradable polymer microneedlesin skin. Biomaterials.

[13] McGrath M.G., Vucen S., Vrdoljak A., Kelly A., O’Mahony C., Crean A.M., Moore A. (2014). Production of dissolvable microneedles using an atomised spray process: Effect of microneedle composition on skin penetration. Eur. J. Pharm. Biopharm..

[14] Choi C.K., Lee K.J., Youn Y.N., Jang E.H., Kim W., Min B.K., Ryu W.H. (2013). Spatially discrete thermal drawing of biodegradable microneedles for vasculardrug delivery. Eur. J. Pharm. Biopharm..

[15] Mellott M.B., Searcy K., Pishko M.V. (2001). Release of protein from highly cross-linked hydrogels of poly(ethylene glycol) diacrylate fabricated by UV polymerization. Biomaterials.

[16] Di Matteo A., Di Palma V., Bevilacqua M.F., Cimmino A. (2014). Biosensor. U.S. Patent.

[17] Hahn M.S. (2010). Patterning of PEG-based hydrogels—Engineering spatial complexity. Mater. Matters.

[18] Politi J., Spadavecchia J., Iodice M., de Stefano L. (2015). Oligopeptide-heavy metal interaction monitoring by hybrid gold nanoparticle based assay. Analyst.

[19] Nada A.M.A., Dawy M., Salama A.H. (2004). Dielectric properties and ac-conductivity of cellulose polyethylene glycol blends. Mater. Chem. Phys..

[20] Larrañeta E., Moore J., Vicente-Pérez E.M., González-Vázquez P., Lutton R., Woolfson A.D., Donnelly R.F. (2014). A proposed model membrane and test method for microneedle insertion studies. Int. J. Pharm..

[21] Mack C. (2008). Fundamental Principles of Optical Lithography: The Science of Microfabrication.

[22] Kochhar J.S., Goh W.J., Chan S.Y., Kang L. (2013). A simple method of microneedle array fabrication for transdermal drug delivery. Drug Dev. Ind. Pharm..

[23] Dutta J. (2012). Synthesis and characterization of γ-irradiated PVA/PEG/CaCl2 hydrogel for wound dressing. Am. J. Chem..

[24] Revzin A., Tompkins R.G., Toner M. (2003). Surface engineering with poly(ethylene glycol) photolithography to create high-density cell arrays on glass. Langmuir.

[25] Liu Y., Liu Y., Matharu Z., Rahimian A., Revzin A. (2015). Detecting multiple cell-secreted cytokines from the same aptamer-functionalized electrode. Biosens. Bioelectron..

[26] Chaudhri B.P., Ceyssens F., de Moor P., Hoof C.V., Puers R. (2010). A high aspect ratio SU-8 fabrication technique for hollow microneedles for transdermal drug delivery and blood extraction. J. Micromech. Microeng..

[27] Yoshida K., Lewinsky I., Nielsen M., Hylleberg M. (2007). Implantation mechanics of tungsten microneedles into peripheral nerve truks. Med. Biol. Eng. Comput..

[28] Yang S.Y., O’Cearbhaill E.D., Sisk G.C., Park K.M., Cho W.K., Villiger M., Bouma B.E., Pomahac B., Karp J.M. (2013). A bio-inspired swellable microneedle adhesive for mechanical interlocking with tissue. Nat. Commun..

[29] Browning M.B., Cereceres S.N., Luong P.T., Cosgriff-Hernandez E.M. (2014). Determination of the *in vivo* degradation mechanism of PEGDA hydrogels. J. Biomed. Mater. Res. Part A.

